# Differential diagnostic value of *P* wave dispersion and QT interval dispersion between psychogenic pseudosyncope and vasovagal syncope in children and adolescents

**DOI:** 10.1186/s13052-025-01864-5

**Published:** 2025-01-23

**Authors:** Zifeng Huang, Yi Xu, Shuo Wang, Ping Liu, Xingfang Zen, Yuwen Wang, Fang Li, Runmei Zou, Cheng Wang

**Affiliations:** 1 Department of Pediatric Cardiovasology, Children’s Medical Center, The Second Xiangya Hospital, Central South University, No.139 Renmin Middle Road, Changsha, 410011 Hunan China; 2 Department of Pediatrics, Xiangya Hospital, Central South University, Changsha, 410008 Hunan China

**Keywords:** Psychogenic pseudosyncope, Vasovagal syncope, *P* wave dispersion, QT interval dispersion, Children, Adolescent

## Abstract

**Background:**

Both psychogenic pseudosyncope (PPS) and vasovagal syncope (VVS) in children and adolescents are diseases of transient loss of consciousness. It is difficult to distinguish them clinically. This paper will study the differential diagnostic value of *P* wave dispersion (Pd) and QT interval dispersion (QTd) between PPS and VVS.

**Methods:**

The 31 children with PPS and 40 children with VVS from July 2014 to November 2023 were enrolled as the study group. Meanwhile, 30 healthy children who underwent a physical examination at the same hospital were matched to the control group. *P* wave duration and QT interval in the 12-lead electrocardiogram were measured at the baseline.

**Results:**

(1) Comparison between groups: ① The Pd, corrected *P* wave dispersion (Pcd), QTd, and corrected QT interval dispersion (QTcd) in PPS group were significantly higher than those in control group (*P* < 0.05). The minimum *P* wave duration (Pmin) and corrected *P* wave duration (Pcmin) in PPS group were significantly lower than those in control group (*P* < 0.05). There were no significant differences in maximum *P* wave duration (Pmax), corrected maximum P-wave duration (Pcmax), maximum QT interval (QTmax), minimum QT interval (QTmin), corrected maximum QT interval (QTcmax), and corrected minimum QT interval (QTcmin) between PPS group and control group (*P* > 0.05). ② The Pd, Pcd, QTd, and QTcd in VVS group were significantly higher than those in control group (*P* < 0.05). The Pmin, Pcmin, and QTcmin in VVS group were significantly lower than those in control group (*P* < 0.05). There were no significant differences in Pmax, Pcmax, QTmax, QTmin, and QTcmax between VVS group and control group (*P* > 0.05). ③ The Pmax, Pd, QTmax, QTd, and QTcd in PPS group were significantly lower than those in VVS group (*P* < 0.05). There were no significant differences in Pmin, Pcmax, Pcmin, Pcd, QTmin, QTcmax, and QTcmin between PPS group and VVS group (*P* > 0.05). (2) ROC curve: Pmax, Pd, QTmax, QTd, and QTcd had a certain differential diagnostic value between PPS and VVS in children and adolescents (*P* < 0.05). QTd had the largest area under curve (0.735), with a sensitivity of 85.00% and a specificity of 53.30% at the cut off value of ≥ 28.11 ms for VVS diagnosis.

**Conclusions:**

In children and adolescents, electrocardiogram parameters such as Pmax, Pd, QTmax, QTd, and QTcd all possess predictive value in differentiating between PPS and VVS. Among them, QTd has the greatest differential diagnostic value.

## Background

Syncope is characterized by a rapid onset of transient loss of consciousness (TLOC) due to transient global cerebral hypoperfusion, which is accompanied by a decrease or loss of muscle tone required to maintain posture [1]. Syncope is a common emergency in children and adolescents, with approximately 20% of males and 50% of females having experienced syncope at least once [2]. Hu et al. [3] conducted a questionnaire survey on 4,352 children and adolescents aged 2 to 18 in Changsha and revealed a syncope incidence of 17.37%, with higher rates observed in adolescence compared to school age and preschool age (28.85% vs. 8.32% vs. 2.71%, *P* < 0.05). Neurally mediated syncope (NMS) is the common cause of syncope in children, with vasovagal syncope (VVS) accounting for approximately 60–70% [2]. In recent years, as a result of detailed research into the etiology of syncope and an increased focus on mental health, psychogenic pseudosyncope (PPS), which is attributed to psychological factors, has received increasing attention. PPS is an apparent loss of consciousness in the absence of impaired cerebral perfusion [4]. However, the similarity of clinical manifestations between PPS and VVS and the lack of understanding of PPS among clinicians make the diagnosis of PPS still challenging. In a study of both adults and children, up to 50% of PPS were misdiagnosed as VVS [5]. The head-up tilt test (HUTT) is currently an important diagnostic test for differentiating PPS from VVS [6], but it can cause several complications such as psychological fear, severe cardiac arrhythmias, transient aphasia, and convulsions during HUTT [7–10]. Therefore, it is significantly important to explore other non-invasive and simple indicators or methods in the diagnosis and differentiation of PPS.

The 12-lead electrocardiogram (ECG) is a classic clinical test that reflects the electrical activity of cardiomyocytes and is widely used in the ancillary diagnosis of various diseases because its non-invasive, straightforward, and cost-effective nature. The *P* wave is a waveform generated during depolarization of the left and right atria, with the first half representing right atrial depolarization and the second half representing left atrial depolarization. The *P* wave dispersion (Pd) refers to the difference between the maximum *P* wave duration (Pmax) and the minimum *P* wave duration (Pmin) in a 12-lead ECG, which is a sign of inhomogeneous electrical activity in the atria, and it is an important indicator for the clinical assessment and prediction of atrial arrhythmias. Prolonged Pmax serves as a marker of delayed intra- or inter-atrial conduction, and Pmax as well as Pd can reflect sympathetic activity [11, 12]. The QT interval represents the total time between ventricular depolarization and repolarization, and the QT interval dispersion (QTd) refers to the difference between the maximum QT interval (QTmax) and the minimum QT interval (QTmin) in a 12-lead ECG, reflecting the inhomogeneous repolarization in the ventricle. It is clinically valuable in predicting the risk of malignant arrhythmias and cardiovascular disease. To some certain extent, QTmax may reflect the influence of the sympathetic nervous system on ventricular repolarization, and there is evidence suggesting that QTmax may also serve as an indicator of cardiac death additionally [13, 14]. Corrected *P* wave dispersion (Pcd) and corrected QT interval dispersion (QTcd) improve the accuracy of the results by correcting for heart rate. Currently, there are few reports on the differentiation between PPS and VVS by Pd and QTd in children and adolescents. This study aims to study the differential diagnostic value of Pd and QTd between PPS and VVS in children and adolescents.

## Methods

### Study subjects

The 31 children with PPS (20 males, 11 females, age 12.47 ± 3.17 years old) diagnosed in Pediatric Cardiovascular Ward or Pediatric Psychiatric Ward and 40 children with VVS (19 males, 21 females, age 11.21 ± 2.57 years old) diagnosed in Pediatric Cardiovascular Ward, The Second Xiangya Hospital, Central South University from July 2014 to November 2023 were enrolled as the study group. Patients underwent a comprehensive evaluation, including detailed medical history, physical examination, imaging tests (such as chest X-ray, electroencephalogram, echocardiography, head CT or MRI, etc.), blood tests (cardiac enzymes, electrolytes, fasting plasma glucose, immunological markers, etc.), 12-lead ECG, 24-hour Holter ECG, and 24-hour ambulatory blood pressure monitoring, etc., were conducted. After exclusion of organic cardiovascular and cerebrovascular diseases, metabolic disorders, and immune diseases in all cases, PPS and VVS were diagnosed. Meanwhile, 30 healthy children (16 males, 14 females, age 11.17 ± 1.94 years old) who underwent a physical examination in Department of Child Health Care of the same hospital during the same period were matched to the control group. HUTT is a noninvasive examination which has been approved by the Ethics Committee of The Second Xiangya Hospital, Central South University (2014-012).

The calculation of sample size: Based on the empirical method, this study obtained the approximate required sample size after comparing previous similar studies with positive results [15–17].

### Methodology of HUTT [18]

HUTT was performed in the morning in a quiet environment with dim lighting, a comfortable room temperature, and no distractions. Subjects fasted for at least 4 h before the test, stopped any vasoactive medication for at least 5 half-lives, and avoided beverages that could affect autonomic nervous system function (e.g., coffee). Subjects and their guardians were informed of the preparations, procedures, and the potential risks and its appropriate solutions before the test, and the informed consent was signed by guardians. After bladder emptying, subjects laid on the tilt table (SHUT-100 A tilt test monitoring software system, Beijing Standley Technology Co., LTD) for 10 min with chest and knee bands fixed to avoid falling and flexion, and the baseline heart rate (HR), blood pressure (BP), and 12-lead ECG were recorded. Within 15 s, the patients were converted to a 60° head-up and foot down tilt position. The whole test lasted for 45 min. During HUTT, BP, HR, ECG, and clinical manifestations were continuously monitored and recorded until the test was terminated after a positive reaction, and the supine position was restored within 10 s. If there was no positive response to the basic HUTT, subjects underwent the sublingual nitroglycerin provoked HUTT (SNHUT), in which 4–6 µg/kg (maximum ≤ 300 µg) of nitroglycerin was administered sublingually and the same position was maintained for a further 20 min, and BP, HR, ECG and clinical manifestations were recorded until a positive response was obtained. The result was negative if there was no positive response during SNHUT.

Standards for a positive response of VVS [18]: syncopal episodes or presyncopal signs together with any of the following responses in the HUTT are considered positive responses:

systolic blood pressure (SBP) ≤ 80 mmHg (1 mmHg = 0.133 kPa) or diastolic blood pressure (DBP) ≤ 50 mmHg or mean pressure decrease ≥ 25%; 2) HR < 75 beats/min for 4 to 6 years old children, < 65 beats/min for > 6 to 8 years old children, and < 60 beats/min for those > 8 years old children and adolescents; 3) ECG showing sinus arrest or junctional escape rhythm; 4) atrioventricular block (II or III degree) or cardiac arrest ≥ 3 s.

Classifications of VVS [18]: (1) vasoinhibitory type VVS (VVS-VI): a significant decrease in BP without obvious HR reduction; (2) cardioinhibitory type VVS (VVS-CI): a marked HR decrease without obvious BP decrease; (3) mixed type VVS (VVS-M): both an HR and BP obvious decrease.

### Diagnosis of PPS

The study subjects of PPS were diagnosed according to Diagnostic and Statistical Manual of Mental Disorders, fifth Edition (DSM-5) [19].

### Electrocardiogram

A supine ECG was performed on the day before the HUTT. Before ECG recording, 5 half-lives of cardiovascular active drugs were stopped. Subjects were asked to maintain in the supine position, and the 12-lead ECG was recorded by MAC800 ECG comprehensive automatic analyzer [GE HealthCare (China) Co., LTD]. A gain of 1 mV = 10 mm and a paper speed of 25 mm/s were used, and no filtering device was used for sampling. The computer automatically measured the various waveforms on ECG with human intervention as necessary. The three clear cardiac cycles of sinus rhythm were measured and averaged.

Measurement of Pd: Using the initial position of Q wave as the reference level to measure Pmax and Pmin, and Pd was generated by calculating the difference between Pmax and Pmin. Corrected Pmax (Pcmax) and corrected Pmin (Pcmin) were calculated using Bazett formula, and Pcd was generated by calculating the difference between Pcmax and Pcmin.

Measurement of QTd: Using the initial position of Q wave as the reference level to measure QTmax and QTmin, and QTd was generated by calculating the difference between QTmax and QTmin. Corrected QTmax (QTcmax) and corrected QTmin (QTcmin) were calculated using Bazett formula, and QTcd was generated by calculating the difference between QTcmax and QTcmin. The endpoint of T wave determined by [20]: (1) T wave back to reference line; (2) tangent point between T wave and U wave; (3) intersection of two-phase T wave finally returning to reference line.

### Statistical analysis

Statistical analysis was conducted with SPSS 26.0 software (IBM, New York, USA). Measurement data were described as $\left( {\overline x \pm s} \right)$ or median (25th percentile, 75th percentile). The differences of normal distribution data between groups were compared using the *t*-test, and the differences of non-normally distributed data between groups were compared using the Mann-Whitney *U* test. The categorical variables were described by frequency and constituent ratio, and comparisons between groups were made using chi-square tests or Fisher’s precision probability test. The receiver-operating characteristic (ROC) curve was performed to determine whether related parameters of *P* wave and QT interval held predictive value in distinguishing between PPS and VVS. A *p*-value < 0.05 was considered significant.

## Results

### Demographic features

There were no significant differences in age (*t* = -1.920, *P* = 0.060) and sex (*χ*^*2*^ = 0.788, *P* = 0.375) between PPS group and control group. There were no significant differences in age (*Z* = -0.421, *P* = 0.673) and sex (*χ*^*2*^ = 0.233, *P* = 0.629) between VVS group and control group. There were no significant differences in age (*Z* = -1.513, *P* = 0.130) and sex (*χ*^*2*^ = 2.043, *P* = 0.153) between PPS group and VVS group.

### Comparison of pd, Pcd, QTd, and QTcd between groups

The Pd, Pcd, QTd, and QTcd in PPS group were significantly higher than those of control group (*P* < 0.05). The Pmin and Pcmin in PPS group were significantly lower than those of control group (*P* < 0.05). There were no significant differences in Pmax, Pcmax, QTmax, QTmin, QTcmax, and QTcmin between PPS group and control group (*P* > 0.05, Table 1). The Pd, Pcd, QTd, and QTcd in VVS group were significantly higher than those of control group (*P* < 0.05). The Pmin, Pcmin, and QTcmin in VVS group were significantly lower than those of control group (*P* < 0.05). There were no significant differences in Pmax, Pcmax, QTmax, QTmin, and QTcmax between VVS group and control group (*P* > 0.05, Table 2). The Pmax, Pd, QTmax, QTd, and QTcd in PPS group were significantly lower than those of VVS group (*P* < 0.05). There were no significant differences in Pmin, Pcmax, Pcmin, Pcd, QTmin, QTcmax, and QTcmin between PPS group and VVS group (*P* > 0.05, Table 3).

**Table 1:** Comparison of pd, Pcd, QTd and QTcd between PPS group and control group [$\left( {\overline x \pm s} \right)$ or *M* (*P*25, *P*75), ms]

Variable	Control group	PPS group	t/Z	*P* value
Pmax	89.22(85.40, 91.56)	84.25(81.75, 91.05)	-1.959	0.050
Pmin	73.03(68.57, 77.23)	64.17(61.09, 68.77)	-4.014	<0.001
Pd	16.15 ± 4.14	20.33 ± 5.17	-3.457	0.001
Pcmax	102.39 ± 11.32	99.36 ± 11.66	1.022	0.311
Pcmin	82.90(77.19, 91.06)	74.00(68.39, 81.11)	-3.437	0.001
Pcd	18.70 ± 5.35	23.36 ± 5.96	-3.190	0.002
QTmax	389.36(366.99, 401.27)	382.80(368.20, 395.88)	-0.621	0.535
QTmin	362.30 ± 24.34	355.11 ± 23.44	1.165	0.249
QTd	24.60 ± 5.93	29.01 ± 8.96	-2.251	0.028
QTcmax	445.10 ± 26.82	441.28 ± 26.95	0.550	0.584
QTcmin	416.65 ± 24.80	407.85 ± 25.14	1.364	0.178
QTcd	28.45 ± 7.62	33.47 ± 10.78	-2.080	0.042

PPS: psychogenic pseudosyncope; Pmax: maximum P-wave duration; Pmin: minimum P-wave duration; Pd: P-wave dispersion; Pcmax: corrected maximum P-wave duration; Pcmin: corrected minimum P-wave duration; Pcd: corrected P-wave dispersion; QTmax: maximum QT-interval; QTmin: minimum QT-interval; QTd: QT-interval dispersion; QTcmax: corrected maximum QT-interval; QTcmin: corrected minimum QT-interval; QTcd: corrected QT-interval dispersion

**Table 2:** Comparison of pd, pcd, QTd and QTcd between VVS group and control group [$\left( {\overline x \pm s} \right)$ or *M* (*P*25, *P*75), ms]

Variable	Control group	VVS group	t/Z	*P* value
Pmax	88.67 ± 6.01	90.46 ± 8.40	-0.990	0.326
Pmin	72.52 ± 5.12	65.84 ± 7.19	4.328	<0.001
Pd	15.96(12.19, 19.06)	22.43(19.48, 30.09)	-5.198	<0.001
Pcmax	102.39 ± 11.32	101.65 ± 13.12	0.250	0.803
Pcmin	83.69 ± 8.80	73.87 ± 9.51	4.415	<0.001
Pcd	19.20(14.40, 22.38)	26.69(21.38, 34.03)	-4.658	<0.001
QTmax	386.89 ± 24.02	397.19 ± 33.21	-1.439	0.155
QTmin	362.30 ± 24.34	358.95 ± 28.67	0.514	0.609
QTd	24.60 ± 5.93	38.24 ± 11.97	-6.258	<0.001
QTcmax	445.10 ± 26.82	444.08 ± 29.82	0.140	0.883
QTcmin	416.65 ± 24.80	401.31 ± 24.39	2.586	0.012
QTcd	28.45 ± 7.62	42.78 ± 13.58	-5.598	<0.001

PPS: psychogenic pseudosyncope; Pmax: maximum P-wave duration; Pmin: minimum P-wave duration; Pd: P-wave dispersion; Pcmax: corrected maximum P-wave duration; Pcmin: corrected minimum P-wave duration; Pcd: corrected P-wave dispersion; QTmax: maximum QT-interval; QTmin: minimum QT-interval; QTd: QT-interval dispersion; QTcmax: corrected maximum QT-interval; QTcmin: corrected minimum QT-interval; QTcd: corrected QT-interval dispersion

**Table 3:** Comparison of pd, Pcd, QTd and QTcd between PPS group and VVS group [$\left( {\overline x \pm s} \right)$ or *M* (*P*25, *P*75), ms]

Variable	PPS group	VVS group	t/Z	*P* value
Pmax	84.25(81.75, 91.05)	89.89(86.18, 95.46)	-2.492	0.013
Pmin	64.17(61.09, 68.77)	64.96(59.96, 69.58)	-0.107	0.915
Pd	20.27(16.89, 23.67)	22.43(19.48, 30.09)	-2.415	0.016
Pcmax	99.36 ± 11.66	101.65 ± 13.12	0.755	0.453
Pcmin	74.00(68.39, 81.11)	73.52(66.85, 79.88)	-0.344	0.731
Pcd	24.27(18.67, 27.32)	26.69(21.38, 34.03)	-1.964	0.050
QTmax	382.80(368.20, 395.88)	394.61(376.88, 422.89)	-2.124	0.034
QTmin	355.11 ± 23.44	358.95 ± 28.67	0.599	0.551
QTd	29.01 ± 8.96	38.24 ± 11.97	3.540	0.001
QTcmax	441.28 ± 26.95	444.08 ± 29.82	0.405	0.687
QTcmin	407.85 ± 25.14	401.31 ± 24.39	-1.097	0.277
QTcd	33.47 ± 10.78	42.78 ± 13.58	3.092	0.003

PPS: psychogenic pseudosyncope; Pmax: maximum P-wave duration; Pmin: minimum P-wave duration; Pd: P-wave dispersion; Pcmax: corrected maximum P-wave duration; Pcmin: corrected minimum P-wave duration; Pcd: corrected P-wave dispersion; QTmax: maximum QT-interval; QTmin: minimum QT-interval; QTd: QT-interval dispersion; QTcmax: corrected maximum QT-interval; QTcmin: corrected minimum QT-interval; QTcd: corrected QT-interval dispersion

### ROC curve

**Fig. 1:**
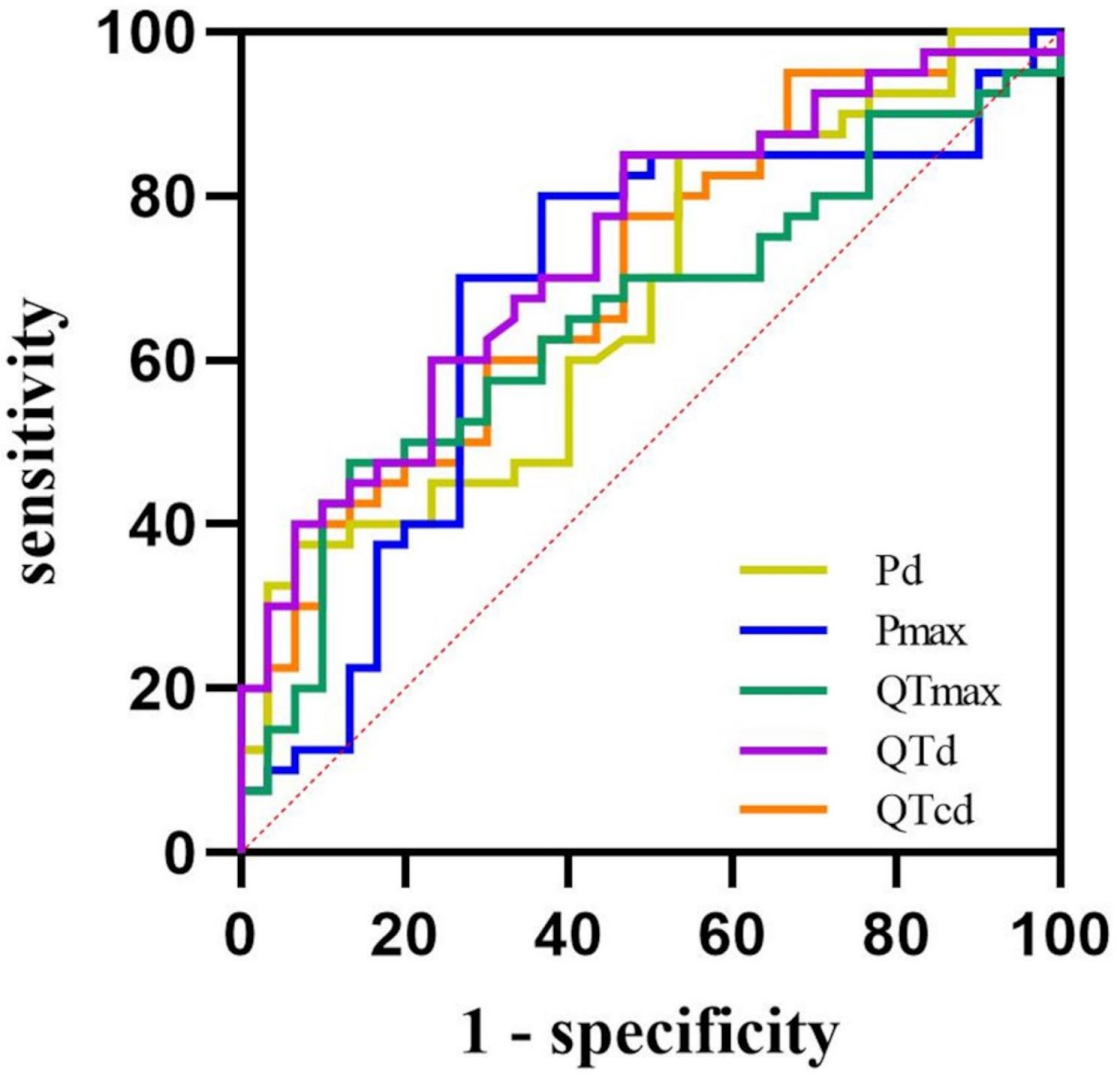
The ROC curve for Pmax, Pd, QTmax, QTd, QTcd predicting the differential diagnosis between PPS and VVS in children and adolescents The Y-axis represents the sensitivity of predicting the effectiveness of Pmax, Pd, QTmax, QTd, QTcd levels between patients diagnosed with PPS or VVS. The X-axis represents the false-positive rate (1-specificity) of the prediction Pmax: maximum P-wave duration; Pd: P-wave dispersion; QTmax: maximum QT-interval; QTd: QT-interval dispersion; QTcd: corrected QT-interval dispersion

The ROC curve was performed to evaluate the predictive value of Pmax, Pd, QTmax, QTd, and QTcd in differentiation between PPS and VVS. Our study found that Pmax, Pd, QTmax, QTd, and QTcd had a certain differential diagnostic value between PPS and VVS in children and adolescents (*P* < 0.05). QTd had the largest area under curve (AUC) (0.735), with a sensitivity of 85.00% and a specificity of 53.30% at the cut off value of ≥ 28.11 ms for VVS diagnosis (Table 4; Fig. 1).

**Table 4:** The value of Pmax, pd, QTmax, QTd, QTcd for differential diagnosis of PPS and VVS

Variable	AUC	*P* value	95% CI	Cutt off value (ms)	Youden index	Sensitivity (%)	Specificity (%)
Pmax	0.675	0.013	0.541∼0.809	85.98	0.433	80.00	63.30
Pd	0.670	0.016	0.543∼0.796	18.85	0.317	85.00	46.70
QTmax	0.649	0.034	0.520∼0.779	399.41	0.342	47.50	86.70
QTd	0.735	0.001	0.618∼0.851	28.11	0.383	85.00	53.30
QTcd	0.698	0.005	0.576∼0.821	32.61	0.308	77.50	53.30

AUC: area under curve; CI: confidence interval; Pmax: maximum P-wave duration; Pd: P-wave dispersion; QTmax: maximum QT-interval; QTd: QT-interval dispersion; QTcd: corrected QT-interval dispersion

## Discussion

VVS is one of the most common causes of syncope in children and adolescents, with the pathogenesis involving either abnormal autonomic reflex regulation or autonomic dysfunction [21]. The autonomic nervous system plays an important role in maintaining homeostasis in human body and it is closely intertwined with the cardiovascular system. The sympathetic nerve and vagal nerve jointly innervate the atria, ventricles, coronary arteries, and peripheral resistance vessels, etc., and ECG waveforms can demonstrate the effects of the interaction between the sympathetic nerve and vagal nerve on the heart. Catecholamines released from the postganglionic sympathetic neuron activate alpha- and beta-adrenergic receptors on cardiomyocyte membranes, while the vagus nerve releases acetylcholine, which acts on muscarinic acetylcholine receptors, both of which influence ion channels and ECG waveforms through their neurotransmitters. Activation of the sympathetic nervous system results in increased HR, increased *P* wave amplitude, shortened PR interval and QRS duration, and a flattened or inverted T wave. Conversely, activation of the vagus nerve results in a decreased HR, decreased *P* wave amplitude, prolonged PR interval and QRS duration, and a peaked T-wave.

The *P* wave reflects the depolarization process of the atrial myocytes, which is the sum of the depolarization vectors from both the left and right atria. The autonomic nervous system can influence the velocity and time of electrical impulses conduction in the atria, and the impact of the autonomic nervous system on the atria is more pronounced due to the richer distribution of nerve endings in the atria compared to the ventricles [22]. Sympathetic excitation shortens the action potential duration in atrial myocytes, increases the slope of phase 0, shortens the refractory period, increases automaticity and triggered activity. Vagal excitation increases the amplitude of the maximum diastolic potentials and action potentials, shortens the action potential duration, reduces automaticity, leading to shorter refractory period, slower conduction, and susceptibility to intra-atrial micro-reentry. Therefore, Pmax and Pd may be altered due to autonomic dysfunction. Autonomic dysfunction, particularly increased sympathetic nerve excitability, notably prolongs both Pmax and Pd [23]. Köse et al. [24] found that Pmax and Pd of HUTT-positive patients were significantly greater than those in the HUTT-negative and healthy controls (*P* < 0.05), suggesting that Pd was an early sign of cardiac autonomic dysfunction in children with NMS. Wang et al. [25] retrospectively analyzed Pd and Pcd in 43 children and adolescents with VVS-CI as well as 43 healthy controls and found that compared with control group, Pd and Pcd in VVS group were significantly higher (*P* < 0.05), with the greatest diagnostic value for VVS-CI at the cut off value of Pd ≥ 27.42 ms (AUC = 0.918, sensitivity = 95.35%, specificity = 69.77%), indicating that autonomic dysfunction in children with VVS leads to prolonged Pd. Our study found that Pmax and Pd in VVS group were significantly higher than those in PPS group (*P* < 0.05), which showed that children with VVS exhibited autonomic dysfunction increased sympathetic excitability resulting in prolonged Pmax and Pd. Additionally, our study also found that Pd, Pcd in PPS group were significantly higher and Pmin, Pcmin were significantly lower than those in control group (*P* < 0.05), considering that it might be associated with the fact that atrial electrical activity was impacted due to anxiety among certain patients with PPS [26]. Uyarel et al. [27] reported that higher levels of individual anxiety correlate with increased Pmax and Pd (*P* < 0.05). Yavuzkir et al. [28] reported that Pmax, Pmin, and Pd in panic disorder, a type of anxiety disorder, were significantly higher than those in healthy controls (*P* < 0.05). This is associated with elevated catecholamine levels due to increased sympathetic activity in individuals experiencing anxiety [29].

The QT interval represents the total duration of ventricular depolarization and repolarization, observed on the surface ECG as the time from the beginning of the QRS complex to the end of the T wave. The autonomic nervous system indirectly modulates the QT interval, with sympathetic nerve activation accelerating the HR and shortening the QT interval, and vagal activation slowing the HR and prolonging the QT interval [30]. QTd refers to the difference between QTmax and QTmin in a 12-lead ECG, reflecting the inhomogeneous repolarization in the ventricle due to the uneven distribution of M cells in the ventricular wall causing transmural and spatial dispersion of myocardial repolarization, which is shown on the surface ECG as QTd [31]. Karataş et al. [32] found that compared to control group, the QTd and QTcd were significantly higher in HUTT-positive response group (*P* < 0.05), which suggested that QTd and QTcd were prolonged due to autonomic dysfunction in children with syncope during HUTT, and believed that QTd held a predictive value for the positive response of HUTT. Liu et al. [33] reported that QTd and QTcd in children with VVS-CI were longer than those in healthy controls (*P* < 0.05) and considered that QTd had a good estimation value in the diagnosis of VVS-CI in children and adolescents. There are few reports of difference in QTd between PPS and VVS in children and adolescents currently. Zhang et al. [34] found that QTd in VVS was significantly higher than those in PPS (*P* < 0.05), and in a scoring model which consisted of syncope duration, upright posture as inducement as well as QTd, a cut off score of ≥ 3 points yielded a sensitivity and specificity of 91.3% and 76.9% respectively for the indication of PPS, with the AUC of 0.909, and believed that this scoring model including QTd was contributed in initial differentiation between PPS and VVS. Our study showed that QTmax, QTd, and QTcd in VVS group were significantly higher than those in PPS group (*P* < 0.05), indicating that QTmax, QTd, and QTcd might have a certain differential diagnostic value between PPS and VVS. Moreover, our study also found that QTd and QTcd in PPS group were significantly higher than those in control group (*P* < 0.05), which may be attributed to the fact that children with PPS are likely to have comorbid anxiety and depression [26]. Piccirillo et al. [35] reported that QTd was significantly higher in patients with anxiety disorder than those in healthy controls (*P* < 0.05), and QTd was positively correlated with the severity of anxiety symptoms, considering an association with sympathetic hyperactivity in patients with anxiety disorders. Nahshoni et al. [15] observed 18 patients with major depressive disorder (MDD) and 9 healthy controls and found that compared to healthy group, QTd and QTcd in MDD group were significantly higher (*P* < 0.05), showing that there may be an increase in sympathetic activity and a decrease in vagal activity in patients with MDD.

In addition, there was one case of anxiety and two cases of depression in the PPS group. There were no children with anxiety and depression in the VVS group and control group. Although previous studies have found an association between anxiety and depression and Pd and QTd, the number of children with anxiety or depression was fewer in our study, limiting the potential for the impact. Consequently, we believe that the results of this study are unlikely to be affected by these factors.

## Limitations

Our study was a single-center retrospective study with a relatively small sample size, further prospective, multicenter studies with large sample sizes are needed to improve the accuracy of the result. What’s more, ECG recordings are susceptible to various confounding factors, which can be assessed by ECGs over multiple time periods and with multiple recordings in further studies.

## Conclusions

In children and adolescents, electrocardiogram parameters such as Pmax, Pd, QTmax, QTd, and QTcd all possess predictive value in differentiating between PPS and VVS. Among them, QTd has the greatest differential diagnostic value.

## Data Availability

The original contributions presented in the study are included in the article/supplementary material, further inquiries can be directed to the corresponding author.

## Abbreviations

TLOC: Transient loss of consciousness
NMS: Neurally mediated syncope
HUTT: Head-up tilt test
SNHUT: Sublingual nitroglycerin provoked HUTT
ECG: Electrocardiogram
Pd: *P* wave dispersion
QTd: QT interval dispersion
QTmax: Maximum QT interval
QTmin: Minimum QT interval
PPS: Psychogenic pseudosyncope
VVS: Vasovagal syncope
VVS-VI: Vasoinhibitory type VVS
VVS-CI: Cardioinhibitory type VVS
VVS-M: Mixed type VVS
HR: Heart rate
BP: Blood pressure
